# Trafficking GRK2: Cellular and Metabolic consequences of GRK2 subcellular localization.

**Published:** 2014-04-08

**Authors:** Daniela Sorriento, Michele Ciccarelli, Gaetano Santulli, Maddalena Illario, Bruno Trimarco, Guido Iaccarino

**Affiliations:** 1 Department of Advanced Biomedical Science, Federico II University, Naples, Italy;; 2 Department of Medicine and Surgery, University of Salerno, Baronissi (SA), Italy;; 3 Columbia University Medical Center, College of Physicians & Surgeons, New York Presbyterian Hospital - Manhattan New York, NY, USA;; 4 Department of Traslational Medical Science, Federico II University of Naples, Naples, Italy;; 5 IRCCS Multimedica, Milano, Italy.

**Keywords:** G protein coupled receptor kinases, mitochondria, energy, cell metabolism

## Abstract

G protein coupled receptor kinase 2 (GRK2) has a key role in cellular function by regulating different intracellular mechanisms in a kinase dependent or independent manner. In this review we have dealt with the recently discovered roles of GRK2 in the regulation of cell metabolism. In particular, we have focused on recent findings about the mitochondrial role of GRK2 in the regulation of energy metabolism. Few findings exist about this topic that all concur to identify a mitochondrial localization of GRK2, leading to the rising of the following question: is GRK2 detrimental or advantageous for mitochondrial function? By the review of available literature, a new concept arises about GRK2 role into the cell,which is that of a stress protein acutely activated by cellular stress whose specific subcellular localization, in particular mitochondrial localization, results in compensatory metabolic responses. Thus, the possibility to regulate GRK2 trafficking within the cell is a promising strategy to regulate the adaptative effects of the kinase on cell metabolism.

## I. G protein coupled receptor kinase 2

G protein coupled receptor kinases (GRK) family comprises seven serine/threonine kinases which phosphorylate and regulate G protein coupled receptors (GPCR) (1). GRKs share structural and functional similarities (2): a well-conserved central catalytic domain is flanked by N-terminal domain, that includes a region of homology to regulators of G-protein signaling (RH), with weak homology among the members of the different subfamilies, and C-terminal domain of variable length,which have little or no sequence homology(3, 4). GRKs have different tissue distribution, subcellular localization, and kinase activity regulation(5, 6). They mostly localize at the plasma membrane (7), but recently new subcellular localizations have been suggested for these kinases (5, 8, 9). GRK2 is known as a cytoplasmic protein that translocates to plasma membrane upon GPCR stimulation where it specifically recognizes and phosphorylates agonist-activated GPCRs leading to receptor desensitization (10, 11). Besides its effect on GPCR regulation, in the last decade GRK2 has been proposed as a multi-functional protein which is involved in the regulation of several cellular functions through the phosphorylation of cytosolic substrates or in a phosphorylation-independent manner through protein-protein interaction (12–18). Recently, new functions for this kinase have been evidenced in several conditions (cardiovascular, inflammatory diseases or cancer), characterized by increased levels of GRK2 (10, 19–21). Such a finding suggests that this kinase could be a potential interesting diagnostic marker and/or therapeutic target for many conditions. In this review we will discuss the role of GRK2 in energy metabolism and mitochondrial function.

## II GRK2 and cell metabolism

An emerging role of GRK2 involves its ability to modulate cell metabolism. The first suggestion that GRK2 is involved in cell metabolism derives from the observation that insulin induces up-regulation of the kinase (16), which in turn inhibits insulin signaling and glucose extraction (16, 19, 22). This observation puts GRK2 at the center of the stage as a possible mechanism for insulin resistance. Also, conditions associated to insulin resistance such as diabetes, hypertension or chronic activation of β adrenergic receptor (16), are characterized by elevated GRK2 levels. The implications for the cell metabolism are identified by transgenic studies showing that overexpression of the kinase leads to resistance to insulin(23). We have recently identified the molecular mechanism by which GRK2 regulates insulin signaling. In vitro, insulin induces an increase of GRK2 levels and causes GRK2–IRS1 association in a time-dependent manner (16), phosphorylation of IRS1 in serine/threonine and inhibition of IRS1 tyrosine phosphorylation leading to inhibition of Insulin Receptor signaling. Other Authors have also proposed a Galphaq dependent mechanism for GRK2 in the regulation of insulin signaling (22). If GRK2 upregulation causes insulin resistance, it is legitimate to speculate that its inhibition would have positive effects on cellular metabolism. Confirmations of such evidence derive from models of diabetes. Anis et al. have designed peptide inhibitorsof GRK2 that prevent its binding to the substrate (24). These peptides correct glucose levels when administered in diabetic gerbils. A similar mechanism has been confirmed in mouse cardiac myocytes, where GRK2 causes IRS-1 phosphorylation at serine 307 (25). In spontaneously hypertensive rats, chronic treatment with a similar inhibitor of GRK2 kinase activity, Ant-124, not only leads to an amelioration of the glucose homeostasis and IRS1 tyrosine phosphorylation, but also to the reduction of the blood pressure levels (16). These findings are in agreement to other literature showing that the inhibition of GRK2 clearly delays the reduction of glucose uptake and protects insulin signaling in the heart, preserving cardiac dimension and function (25). This nurtures a novel scenario in which GRK2 inhibition might correct impaired metabolism in those conditions characterized by poor energy utilization by the cell, such as heart failure. In particular, it is known that GRK2 inhibition obtained through means of transgenic expression of the truncated mutant which prevents GRK2 localization on membranes (GRK2-ct) or deletion of GRK2 gene is beneficial for the failing heart. Nevertheless, this benefit is thought to be dependent upon inhibition of beta adrenergic receptors in the heart. We can also speculate that GRK2 inhibition leads to improved cardiac utilization of energy. Indeed, a recent paper shows that in the course of development of heart failure impaired glucose metabolism precedes cardiac contractility in mice with myocardial infarction (25). These findings support the idea that the inhibition of the kinase activity of GRK2 could be a potential target for therapeutics and, as corollary, GRK2 is tout court deleterious for the cell. However, recent evidences challenge this view, as it is emerging that GRK2 exerts different effects within the cell depending on its localization, cell type, stimuli and physio-pathological context (8, 21, 26–28).

## III. GRK2 and energy metabolism

Beside the known localization of GRK2 in plasma membrane and cytosol, recently an unexpected localization of GRK2 within the cell has been shown. Indeed, it has been demonstrated that GRK2 possesses the ability to localize in mitochondria in different cell lines and contexts (8, 29, 30). Such mitochondrial localization suggests a potential role of GRK2 in the regulation of energy metabolism but to date there are only few and apparently contradictory reports on this topic. Trying to sort out the knowledge available, two major evidences are accumulating:

### 1) The conditions that facilitate GRK2 accumulation in the mitochondria

It is becoming clear that in the resting condition, GRK2 is in mitochondria. This acquisition is supported by evidences gathered with different technologies, including western blotting, immunofluorescence, electron microscopy(8, 29–31). What is interesting, is that different conditions can modulate the amount of mitochondrial GRK2 levels, and in particular stressors events can accelerate this process. In macrophages, GRK2 levels in mitochondria increase during inflammation or LPS stimulation, facilitating biogenesis and restoring mitochondrial function (31). Accordingly, Obrenovich et al. showed that in the early pathogenesis of Alzheimer Disease (AD) and in ischemia reperfusion brain injury models,GRK2 accumulates in damaged mitochondria (30). GRK2 localization to mitochondria appears to be a generalized reactive response in different tissues. Indeed, Chen et al. showed that in hearts in vivo and in cultured myocytes, GRK2 localizes into mitochondria after an ischemiareperfusion insult (29). Chen and co-authors also suggest a potential mechanism by which the kinase is able to traffic to mitochondria. In particular, they demonstrate that phosphorylation at residue Ser670 within the carboxyl-terminus of GRK2 by extracellular signal-regulated kinases (ERK) allows GRK2 to bind the heat shock protein 90 (HSP90), which chaperones the kinase to mitochondria (29). Once again, the involvement with HSP90 is supportive for a stress related response put in place by the cell in a fast manner. Although Chen data illustrate the likely mechanism of mitochondrial transport of GRK2, they do not provide any insight on the mechanisms that anchor GRK2 on mitochondria. On this issue, we have demonstrated that GRK2 recognizes several protein partners, which can be phosphorylated within the mitochondria by the kinase, although so far no data are available to distinguish the identities of such partners(8). Mitochondrial fraction analysis suggests that indeed GRK2 co-localizes with proteins of the intermembrane space, although once again there is only one report available(8). If confirmed, these data are consistent with a different mechanism of localization of GRK2 in plasma membrane and in mitochondria. In particular, GRK2 docks on plasma membrane through means of the carboxy-terminal domain, interacting with βγ subunit of G proteins (32). Such mechanism is so well established that therapeutic strategies aimed to reduce GRK2 mediated membrane receptor desensitization are based on truncated mutants of GRK2 that prevent the kinase interaction with Gβγ subunit (GRK2-ct). Interestingly, this mechanism does not appear to be relevant to mitochondrial localization of GRK2 since transgenic overexpression of GRK2-ct that removes GRK2 from plasma membrane does not reduce GRK2 localization in mitochondria. This is evident from our work (31)and is confirmed also in Chen paper(29), where mitochondrial lysates from neonatal rat cardiac myocytes in resting conditions, infected with an adenovirus encoding for GRK2-ct, still presented an unchanged amount of GRK2. Therefore, it is fair to conclude that GRK2-ct is not the mechanism for GRK2 accumulation in mitochondria.

### 2) The role of GRK2 in mitochondria

This issue is probably more controversial, as different experiments performed with different techniques and in different models have showed apparently contradicting results. In particular, we have demonstrated that GRK2 localizes into mitochondria of HEK-293 cells and enhances mitochondrial biogenesis thus leading to an increase of ATP cellular content (8). The overexpression of GRK2 induces the accumulation of the kinase in mitochondria with a consequent increase of mitochondrial biogenesis antagonizing ATP loss after hypoxia/reperfusion (8). A similar finding, obtained with an opposite strategy, shows that GRK2 removal from the skeletal muscle *in vivo* leads to reduced ATP production and impaired tolerance to ischemia (8). Altogether these findings propose a “positive” regulatory role of GRK2 for mitochondrial biogenesis and ATP generation (31).

**Figure 1:**
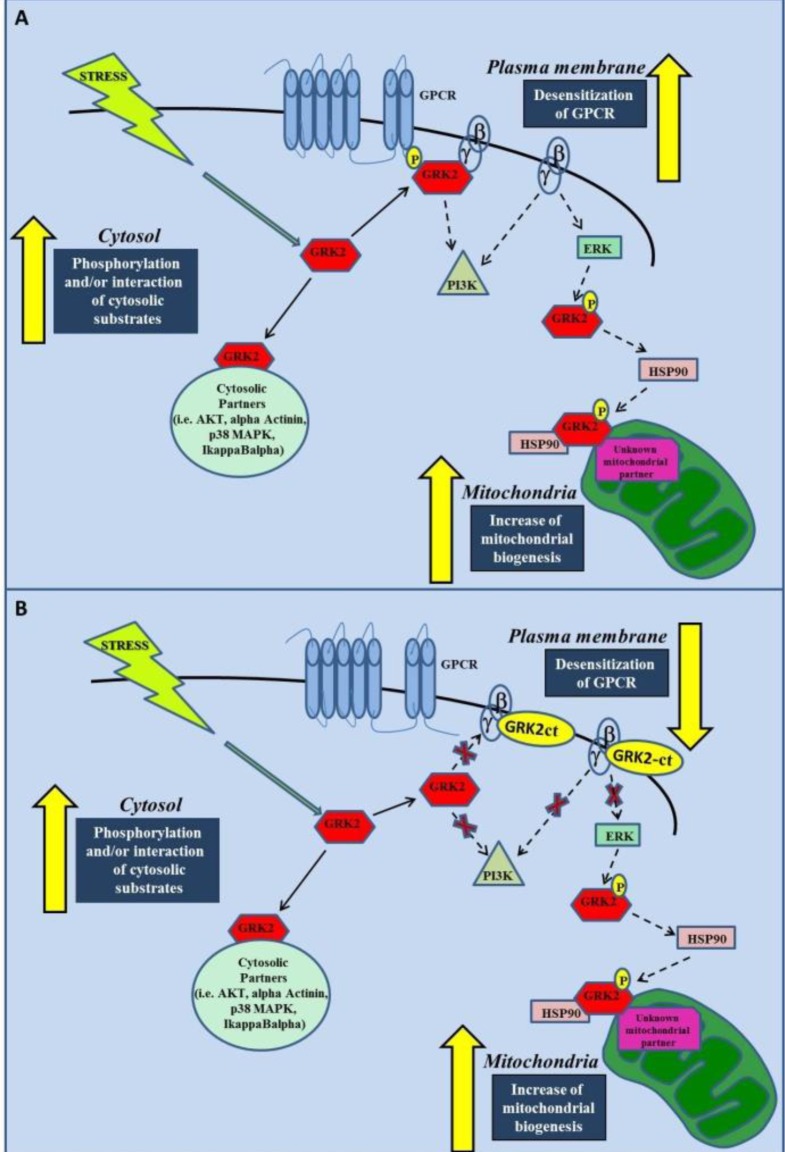
***A–B) Subcellular localization and function of GRK2 in response to stress: effects of GRK2-ct. A)*** *In response to stress, GRK2 moves within different cellular* *compartments* *in a time dependent manner. On plasma membrane, GRK2 interacts with Gβγ subunit, leading to phosphorylation and desensitization of GPCRs, and with* *PI3Kγ, to facilitate its recruitment to the membrane upon agonist stimulation..βγ is also able to transduce signaling* *independently,* *i.e. it activates PI3K and ERK. This latter, on turn, phosphorylates GRK2 in Ser 670 facilitating the interaction with HSP90, which shuttles the kinase towards* *mitochondria.* *Here GRK2 interacts with unknown mitochondrial partners to regulate mitochondrial function. In the cytosol, GRK2 interacts with several proteins (i.e AKT, α–Actinin, p38MAPK, IκBα) to* *regulate* *GPCR independent intracellular signaling.* ***B)*** *GRK2-ct binds βγ and displaces GRK2 from plasma membrane, exerting several effects: 1) it inhibits GRK2-dependent desensitization of GPCRs2) prevent PI3Kγ recruitment to plasma membrane;2)blocks βγ-dependent signaling, such as the activation of ERK, PI3Kγ, and AKT signaling. 4), GRK2-ct makes GRK2 available in other cellular compartments, such as cytosol and mitochondria.*

Using GRK2-ct to inhibit GRK2, Chen et al. show that ischemia/reperfusion injury is no longer able to induce GRK2 accumulation in mitochondria. The final result is the reduction of mitochondrial apoptosis. This finding is interpreted by the Authors as the evidence that GRK2 mitochondrial accumulation causes apoptosis, and that preventing this trafficking favors cell survival. The finding is far from being conclusive and offers alternative interpretations. Indeed, GRK2-ct is well known for its regulatory effects on intracellular signaling, as it blocks Gβγ signaling and activation of ERK(33), which is believed to be the mechanism of GRK2 association to HSP90, but also causes inhibition of pro-apoptotic GRK2-independent signaling such as PI3K pathway(29). From Chen’s study it is not possible to determine the chronologic order of events that takes place when GRK2-ct is expressed in cells subjected to a stress. It might as well be that GRK2-ct, by preventing Gβγ and PI3K related signaling, attenuates the stressor signal within the cell, therefore reducing the reactive stress responses, which includes GRK2 accumulation in mitochondria. This hypothesis needs confirmation in studies where other inhibiting strategies for GRK2 are put in place, i.e. GRK2 silencing or pharmacological inhibition of the catalytic activity, or disruption of GRK2/HSP90 interaction. On the other end, we have demonstrated that in LPS treated macrophages,GRK2-ct is beneficial to the macrophage functionality and survival, by restoring mitochondrial function in a GRK2 dependent manner. Indeed, down-regulation of GRK2 levels by specific siRNA, reduces also GRK2 levels in mitochondria. In this condition, none of the LPS dependent inflammatory phenotypes could be restored by the overexpression of GRK2-ct (31). This clearly suggests that mitochondrial effects of GRK2-ct are strictly dependent on mitochondrial accumulation of GRK2 and confirms the protective role of mitochondrial GRK2 in inflammation probably through its ability to restore mitochondrial biogenesis. Our report clarifies the function of GRK2-ct demonstrating that it is a regulator of GRK2 subcellular localization rather than an inhibitor of the catalytic activity of the kinase, leading to a reduction of its effects on plasma membrane and an increase of its effects in mitochondria (Figure 1).

## Conclusions and perspectives

It is now clear that GRK2 is able to localize in mitochondria (8, 29–31) but the role of the kinase in this organelle is still controversial. Is GRK2 detrimental or advantageous for cell function? It is difficult to find an answer to this question at this stage, giving the multiple roles of GRK2 within the cell. For sure, the perceived role of the kinase within the cell is reshaping. Old evidence together with novel finding propose this kinase as an important adaptive mechanism to stress, such as receptor dependent and independent stimuli. It has been demonstrated that total knock-out of GRK2 in mice is lethal for embryo development (34), thus the generation of transgenic mice with specific organ targeted knock out of GRK2 has been required to perform in vivo studies. This suggests that GRK2 is critical for cell survival. Based on CRE-pLox technique, it has been showed that the removal of GRK2 from specific tissues such as endothelium (35) or cells of the myeloid lineage (36) activates pro-inflammatory phenotypes in mice, thus confirming that GRK2 is protective respect to inflammation. Moreover, it was recently demonstrated that the endothelium-specific knockout of GRK2 is associated with impaired angiogenesis (37). Accordingly, GRK2 deficiency in endothelial cells in vitro increases inflammatory signaling and enhances leukocyte recruitment to activated endothelial cells (38). Furthermore, GRK2 accumulates in mitochondria to restore mitochondrial function both in HEK-293 and RAW264.7 (8, 31), thus suggesting that it is a positive regulator of energy metabolism.

As all the adaptive mechanisms, the effect is helpful at the very beginning, but then, eventually, becomes detrimental for the physiology of the cell. Indeed, several reports suggest a deleterious effect of increased levels of GRK2 in the development of heart failure and insulin resistance when it is increased on plasma membranes (16, 25) and in cardiac cells GRK2 localization in mitochondria seems to be associated with cell death (29). Subcellular localization of the kinase might in the future pose the strategy for selective inhibition of the kinase, and the possibility to modulate GRK2 accumulation within cell organelles could be an useful approach to regulate its negative or positive effects on cell function in a time dependent manner. In this context, GRK2-ct could be an useful strategy to regulate GRK2 subcellular localization and its associated effects. However several issues remain still unsolved: Which is the GRK2 partner to anchor into mitochondria? Which is the mitochondrial signaling that is affected by GRK2? Further studies are needed to answer to these questions to increase the knowledge on the role of GRK2 in mitochondria and elaborate an effective strategy to regulate GRK2 effects within the cell.
